# Prevalence, Distribution, and Genotypes of Adenovirus and Norovirus in the Puzi River and Its Tributaries and the Surrounding Areas in Taiwan

**DOI:** 10.1029/2021GH000465

**Published:** 2021-12-01

**Authors:** Viji Nagarajan, Jung‐Sheng Chen, Bing‐Mu Hsu, Gwo‐Jong Hsu, Jiun‐Ling Wang, Bashir Hussain

**Affiliations:** ^1^ Department of Earth and Environmental Sciences National Chung Cheng University Chiayi County Taiwan; ^2^ Department of Medical Research E‐Da Hospital Kaohsiung Taiwan; ^3^ Division of Infectious Diseases Ditmanson Medical Foundation Chia‐Yi Christian Hospital Chiayi County Taiwan; ^4^ Department of Internal Medicine National Cheng Kung University Hospital Tainan Taiwan; ^5^ Department of Biomedical Sciences National Chung Cheng University Chiayi County Taiwan

**Keywords:** aquatic ecosystem, enterovirus, human and porcine adenoviruses, nested PCR, phylogenetic studies, RT‐PCR

## Abstract

This study investigated the prevalence, distribution, and genotypes of adenoviruses (AdVs) and noroviruses (NoVs) in the Puzi River and surrounding areas in Taiwan. The viruses in the water samples were isolated using the membrane filtration method and the viral nucleic acids were extracted. The RNA of NoVs was reverse‐transcribed into complementary DNA using reverse transcriptase‐polymerase chain reaction. AdVs and NoVs were detected using nested PCR. Genotyping and phylogenetic analyses were performed to identify the various viral genotypes in the water samples. Human adenovirus (HAdVs) and porcine adenovirus (PAdVs) were the predominant genotypes in the water samples. The prevalence of F species HAdVs serotype 41 (79.2%) and C species PAdVs serotype 5 (18.1%) was higher than that of other serotypes. Among NoVs, genogroup GII was more prevalent than GI. In particular, GII.4 (21.2%) and GII.17 (18.2%) were the predominant genotypes, which was consistent with the clinical findings. The prevalence of both AdVs and NoVs was higher in the winter than spring, summer and autumn seasons. AdVs and NoVs detection results were statistically analyzed by investigating their association with water quality indicators. The results revealed that the presence of AdVs was significantly correlated with the heterotrophic bacterial count, total coliform *Escherichia coli*, turbidity, salinity, and dissolved oxygen. Meanwhile, the presence of NoVs was only significantly correlated with temperature, pH, and dissolved oxygen. Microbial pollution sources may include urban runoff and discharge of water from livestock farms situated near the river and tributaries within this region of Taiwan. Future studies should include comparisons of the presence of AdVs and NoVs in these known pollution sources and water quality monitoring of these watersheds, as this will allow potential identification of pollution sources. Additionally, remediation strategies must be developed to minimize viral contamination in the river ecosystem.

## Key Points

- AdVs prevalence is higher in the river than in other sites
- HAdV 41 and PAdV 5 are the dominant genotypes over all sample sites
- Dominant NoVs genotypes are GII.4 and GII.17, they were associated with clinical cases

## 1 Introduction

The deteriorating quality of surface water, which is the major source of drinking water, is a global health concern (Gibson & Schwab, 2011). The major sources of water contamination are natural processes (such as heavy rainfall and runoff) and anthropogenic activities (such as agriculture, construction, and mining). The release of untreated municipal and industrial wastewater into the aquatic ecosystems often results in bacterial and viral contamination, specifically enteric virus contamination (Jean et al., 2006).

The major biological parameters that determine environmental water quality include the presence of enteric viruses (Opere et al., 2020), which pose potential human health risks. The genome of adenoviruses (AdVs), which are the largest known non‐enveloped enteric viruses, comprises a linear double‐stranded DNA (Van Heerden et al., 2005). AdVs have been detected in various water bodies, including oceans, rivers, swimming pools, and wastewater. Human AdVs (HAdVs) are classified into 75 serotypes (HAdV serotypes 1–75) belonging to seven species (HAdV species A–G), while porcine AdVs (PAdVs) are classified into five serotypes (PAdV serotypes 1–5) belonging to three species (PAdV species A–C) (Dhingra et al., 2019; King et al., 2012). Previous studies have reported that the presence of AdVs in the aquatic ecosystems is an indicator of pollution of human origin (Pina et al., 1998). AdVs, which are included in the drinking water contaminant candidate list of the Environmental Protection Agency (EPA), are the etiological agents of various human and animal diseases (EPA, 2016). HAdVs cause respiratory disease, conjunctivitis, and gastroenteritis in children and adults (Jones et al., 2007; Wyn‐Jones et al., 2011). In Taiwan, the prevalence and outbreaks of HAdV‐related gastroenteritis have been increasing annually (Tsou et al., 2011). PAdVs are the etiological agents for gastrointestinal and respiratory diseases in pigs (Fongaro et al., 2015). AdVs are stable under adverse environmental conditions and can survive for a prolonged duration in water, which may be due to the double‐stranded DNA viral genome and utilization of the host cell repair enzymes by the virus (Thurston‐Enriquez et al., 2003). Nested PCR is used for the detection of AdVs in water samples (Allard et al., 2001). To detect AdVs, water samples must be concentrated from large volumes using the adsorption‐elution protocol with membrane filters or the pore size exclusion protocol with ultrafilters (Hill et al., 2007).

Noroviruses (NoVs), which are non‐enveloped viruses with a linear, non‐segmented, single‐stranded RNA, spread through the fecal‐oral route (Glass et al., 2009). Previous studies have reported that NoVs are associated with outbreaks of acute gastroenteritis, nausea, vomiting, and diarrhea (Nguyen et al., 2017). NoVs exhibit enhanced resistance to environmental degradation and disinfectants, which may be attributed to the presence of a non‐fatty lipid membrane (Rutjes et al., 2006). NoVs are classified into seven genogroups (GI to GVII), which are further classified into different genotypes (Atmar et al., 2019). Reverse transcription‐polymerase chain reaction (RT‐PCR) is commonly used to detect NoVs (Vinjé et al., 2003).

Recently, 62.3% of respiratory infections in Taiwan were reported to be associated with the prevalence of HAdVs (Wang et al., 2013). Moreover, hospitalization cases of NoV‐associated acute gastroenteritis have increased by 30% and 11% in children and adults, respectively (Burke et al., 2018). As AdVs and NoVs are waterborne, there is a need to monitor urban river water to determine their prevalence. This study aimed to examine the prevalence, distribution, seasonal distribution, and genetic diversity of AdVs and NoVs. A surveillance study was conducted at the Puzi River and its tributaries, fishing ports, and coastal oyster breeding areas. Additionally, the correlation between water quality indicators and the presence of AdVs and NoVs was investigated. These findings will enhance our understanding of the prevalence, seasonal distribution, and genetic diversity of AdVs and NoVs in the river and coastal ecosystems.

## 2 Materials and Methods

### 2.1 Sampling Locations and Distribution of Sampling Points

Water samples were collected from the Puzi River and its tributaries, the Dongshi fishing port, and the coastal oyster breeding area between December 2015 and November 2016 in the following four seasons: winter (December–February), spring (March–May), summer (June–August), and autumn (September–November). The samples were collected in each of the locations within the given season a day apart in the order of Puzi River tributaries on the first day, Puzi River on the second day, Dongshi fishing port and coastal oyster breeding area on the third day of respective seasons. The Puzi River and its tributaries are mainly utilized for public water supply. Additionally, the water is used for agricultural irrigation, industries, fish farming, animal husbandry and recreational activities. There are many agricultural farms and municipal wastewater treatment plants located in the surrounding area. The Dongshi fishing port water is mainly utilized for the depuration process and the coastal oyster farm area water is used for oyster breeding activities. An overview of the sampling sites is presented in Figure S1 in Supporting Information S1. In total, 96 samples (24 samples per season) were collected from the Puzi River from the following three districts: district A (36/96), upstream region of the livestock and breeding confluence section (PR01‐14); district B (28/96), middle stream town region (PR15‐25); district C (32/96), region adjacent to the estuary (PR26‐34) (Figure S2a in Supporting Information S1). Additionally, 116 samples (29 samples per season) were collected from the outflow of livestock farm wastewater channel (LFWC; 52/116), municipal wastewater treatment plant channel (MWTPC; 12/116), and upstream tributary (UT; 52/116) at 29 locations around the Puzi River (Figure S2b in Supporting Information S1). Furthermore, 12 (three samples per season) samples were collected from the Dongshi fishing port, Chaiyi. Finally, 60 samples (15 samples per season) were collected from the coastal oyster breeding area of the estuary and offshore region of the Puzi River.

### 2.2 Water Sample Collection and Water Quality Examination

Approximately 1 L of water sample was collected at each sampling site and stored in wide‐mouth‐sterilized polypropylene bottles. The samples were stored in an icebox at 4°C and delivered to the laboratory within 8 hr of collection. The sample record form containing sampling, storage, and transportation details were filled out and maintained for future reference.

The water samples were subjected to various quality assessments. The temperature of the water sample was measured immediately after sampling using a thermometer. The pH and conductivity of the water samples were examined using a pH and conductivity meter (D‐24, HORIBA), respectively. Turbidity of the water samples was measured using a turbidimeter (Turb555, WTW). The counts of heterotrophic bacteria were measured using the smear method (E203.56B, 2013). Total coliforms were determined by membrane filtration and the membrane was placed on a coliform agar enhanced selectivity enzyme color medium (E237.52B, 2013) for bacterial enumeration. The samples were incubated at 35 ± 1°C for 22–24 hr. The numbers of *Escherichia coli* (*E. coli*) and total coliforms, which were identified based on the appearance of dark blue/blue‐purple and red colonies, respectively, were counted.

### 2.3 Virus Concentration, Nucleic Acid Extraction, and Reverse Transcription

To enhance virus recovery from the samples, the water samples were concentrated before analysis. Water samples (1 L) were vacuum‐filtered through a 42 mm GN‐6 membrane with a pore size of 0.45 μm (GN‐Metricel PALL) (Huang et al., 2015). Next, the filter paper was placed in 45 mL of phosphate‐buffered saline (PBS, pH 7.2) and kneaded for 5 min with sterile gloves. The eluate was then transferred into centrifuge tubes and centrifuged at 2,600 × g for 30 min (KUBOTA 2420 Compact tabletop centrifuge). Further, 2 mL of the pellet was resuspended in phosphate‐buffered saline (5 mL) at 4°C and subjected to total viral nucleic acid extraction.

Viral nucleic acid (DNA/RNA) extraction was performed with the concentrated water sample using the MagPurix® viral nucleic acid extraction kit ZO02006 and a fully automated MagPurix 12s nucleic acid extraction system (Zisnexts Life Science Corp.). The presence of AdV and NoV nucleic acids was determined.

As NoV is an RNA virus, the RNA must be reverse‐transcribed into complementary DNA (cDNA) before analysis. The extracted RNA was subjected to reverse transcription (RT) using a cDNA RT kit (Bosite Biotechnology Limited Company). The RT reaction mixture comprising 50 μL of RNA and 2.5 μL of random hexamers was heated at 65°C for 5 min to facilitate the opening of the RNA secondary structure. Next, the mixture was incubated with 20 μL of 5X reaction buffer, 2.5 μL of Moloney murine leukemia virus (MMLV) reverse transcriptase, 2.5 μL of RNase inhibitor, 10 μL of 10 mM deoxyribonucleotide triphosphate (dNTP) pre‐Mix and 12.5 μL of diethyl pyrocarbonate‐treated water in ice. RT was performed in a thermal cycler (Px2 Thermal Cycler, Thermo). The PCR conditions were as follows: 25°C for 10 min (random hexamer binding), 42°C for 60 min (RT), and 70°C for 10 min (termination of the reaction) (Hata et al., 2014). cDNA was stored at −20°C until nested PCR analysis.

### 2.4 Nested PCR, Sequencing, and Phylogenetic Analysis

For AdV detection, the nested PCR mixture comprised of 2 μL of DNA template, 1 μL of each of the outer and inner primer sets (0.4 μM), 5 μL of Fast‐Run Tag Master Mix with dye, and 17 μL of PCR‐grade water was prepared. Hex1deg/Hex2deg and neHex3deg/neHex4deg are the outer and inner primer sets used for the detection of AdVs, respectively (Allard et al., 2001). The PCR conditions were as follows: 95°C for 5 min (denaturation), followed by 40 cycles of 95°C for 30 s, 56°C 30 s, and 72°C for 30 s, with a final extension step of 72°C for 5 min, and hold at 4°C (Thermo Px2 Thermal Cycler, Thermo).

To detect NoVs, a 25‐μL nested PCR mixture comprised of 3 μL of cDNA template, 1 μL of each of the outer and inner primers (0.4 μM), 5 μL of Fast‐Run Tag Master Mix with dye, and 12 μL of PCR‐grade water was prepared. COG1F/G1‐SKR and G1‐SKF/G1‐SKR were the outer and inner primer sets used for the detection of NoV GI genotype, respectively (Tajiri‐Utagawa et al., 2009). The first PCR conditions were as follows: 95°C for 4 min (initial denaturation), followed by 40 cycles of 95°C for 30 s, 56°C for 30 s, and 72°C for 1 min, with a final extension step of 72°C for 7 min (final extension), and hold at 4°C. The second PCR conditions were as follows: 95°C for 4 min (denaturation), followed by 40 cycles of 95°C for 30 s, 60°C for 30 s, and 72°C for 1 min, a final extension step of 72°C for 7 min, and hold at 4°C. The outer and inner primer sets for the NoV GII genotype were COG2F/ G2‐SKR and G2‐SKF/ G2‐SKR, respectively (Hata et al., 2014). The first PCR conditions were as follows: 95°C for 4 min (denaturation), followed by 40 cycles of 95°C for 30 s, 56°C for 30 s, and 72°C for 1 min, a final extension step of 72°C for 7 min, and hold at 4°C. The second PCR conditions were as follows: 95°C for 4 min (denaturation), followed by 40 cycles of 95°C for 30 s, 60°C for 30 s, and 72°C for 1 min, a final extension step of 72°C for 7 min, and hold at 4°C.

The PCR products (5 μL) were mixed with 1 μL of DNA‐loading dye and subjected to agarose gel electrophoresis using a 1.5% gel in Tris‐acetate buffer (TAE) at 100 V for 30 min (Apelex P.S 304). The resolved bands were visualized under a UV transilluminator and the images were captured using a gel documentation system (Sankyo Denki G14T8‐AN). The nested PCR products of AdVs and NoVs from the positive samples were sequenced. The PCR products were excised from the gel and purified. The purified nested PCR products were sequenced by Mission Biotech, Taiwan (Applied Biosystems 3730xl DNA Analyzer). The nucleotide sequences were compared using the Basic Local Alignment Search Tool in the National Center for Biotechnology Information gene database. Phylogenetic analysis was performed using Molecular Evolutionary Genetics Analysis (MEGA) software (version 7.0; MEGA software, USA).

### 2.5 Statistical Analysis

All viral and water quality data were analyzed using the statistical software STATISTICA 6.0 (Statsoft). The Mann‐Whitney U test was performed to analyze the correlation between the presence of AdVs and NoVs and water quality indicators.

## 3 Results

### 3.1 Seasonal Detection of Adenovirus and Norovirus

The detection rate of AdVs in the water samples from the Puzi River and its tributaries (UT, LFWC, and MWTPC), Dongshi fishing port, and coastal oyster breeding area in Taiwan are summarized in Table 1. The detection rate of AdVs was the highest in the Puzi River (75%), followed by its tributaries (56%), Dongshi fishing port (41.7%), and coastal oyster breeding area (11.7%). Besides, the detection rate of AdVs was the highest in the Puzi River during the winter in seasonal comparisons made throughout the year (e.g., 91.7%, 75%, 70.8%, and 62.5% in winter, autumn, summer and spring, respectively). In the tributary water samples, the prevalence of AdVs was the highest in autumn (62%), followed by summer (58.6%), winter (55.2%), and spring (41.4%). Additionally, the prevalence of AdVs in the Dongshi fishing port was the highest in autumn and spring (66.7%), followed by winter (33.3%). Compared with other sampling sites, the oyster breeding area exhibited a much lower prevalence of AdVs in three seasons (26.7%, 13.3%, 6.7% in autumn, summer, and spring, respectively). AdVs were not detected in the Dongshi fishing port and oyster breeding area in spring and winter, respectively.

**Table 1:** Seasonal Detection Rates of Adenoviruses (AdVs) and Noroviruses (NoVs) in the Puzi River and Its Tributaries, the Dongshi Fishing Port, and the Coastal Oyster Breeding Area

Sampling site	Total virus detection rate		Seasonal percentage (%) detection							
			Winter		Spring		Summer		Autumn	
	AdVs	NoVs	AdVs	NoVs	AdVs	NoVs	AdVs	NoVs	AdVs	NoVs
Puzi main river (*n* ^a^ = 96)	75%	11.5%	91.7%	45.8%	62.5%	0%	70.8%	0%	75%	0%
Tributaries (*n* ^a^ = 116)	56%	10.3%	55.2%	0%	41.4%	10.3%	58.6%	3.5%	69%	27.6%
Dongshi fishing port (*n* ^a^ = 12)	41.7%	8.3%	33.3%	33.3%	0%	0%	66.7%	0%	66.7%	0%
Coastal oyster breeding area (*n* ^a^ = 60)	11.7%	1.7%	0%	6.7%	6.7%	0%	13.3%	0%	26.7%	0%

^a^ The total collection sample amount is combined from each season and the collection is the same over each season.

The detection rate of NoVs was the highest in the Puzi River (11.5%), followed by its tributaries (10.3%), Dongshi fishing port (8.3%), and coastal oyster breeding area (1.7%) (Table 1). Additionally, seasonal detection rates of NoVs indicated that the prevalence was the highest in winter, followed by autumn. In the winter season, the prevalence of NoVs was the highest in the Puzi River (45.8%), followed by Dongshi fishing port (33.3%) and coastal oyster breeding area (6.7%). The prevalence of NoVs in the tributaries was the highest in autumn (27.6%). However, the prevalence of NoVs in the tributaries was low in spring (10.3%) and summer (3.5%). Additionally, NoVs were not detected in the tributaries in winter. At several sampling sites, including the Puzi River, Dongshi fishing port, and oyster breeding area, NoVs were not detected in the spring and summer seasons.

### 3.2 Distribution of AdVs and NoVs Hotspots

**Figure 1:**
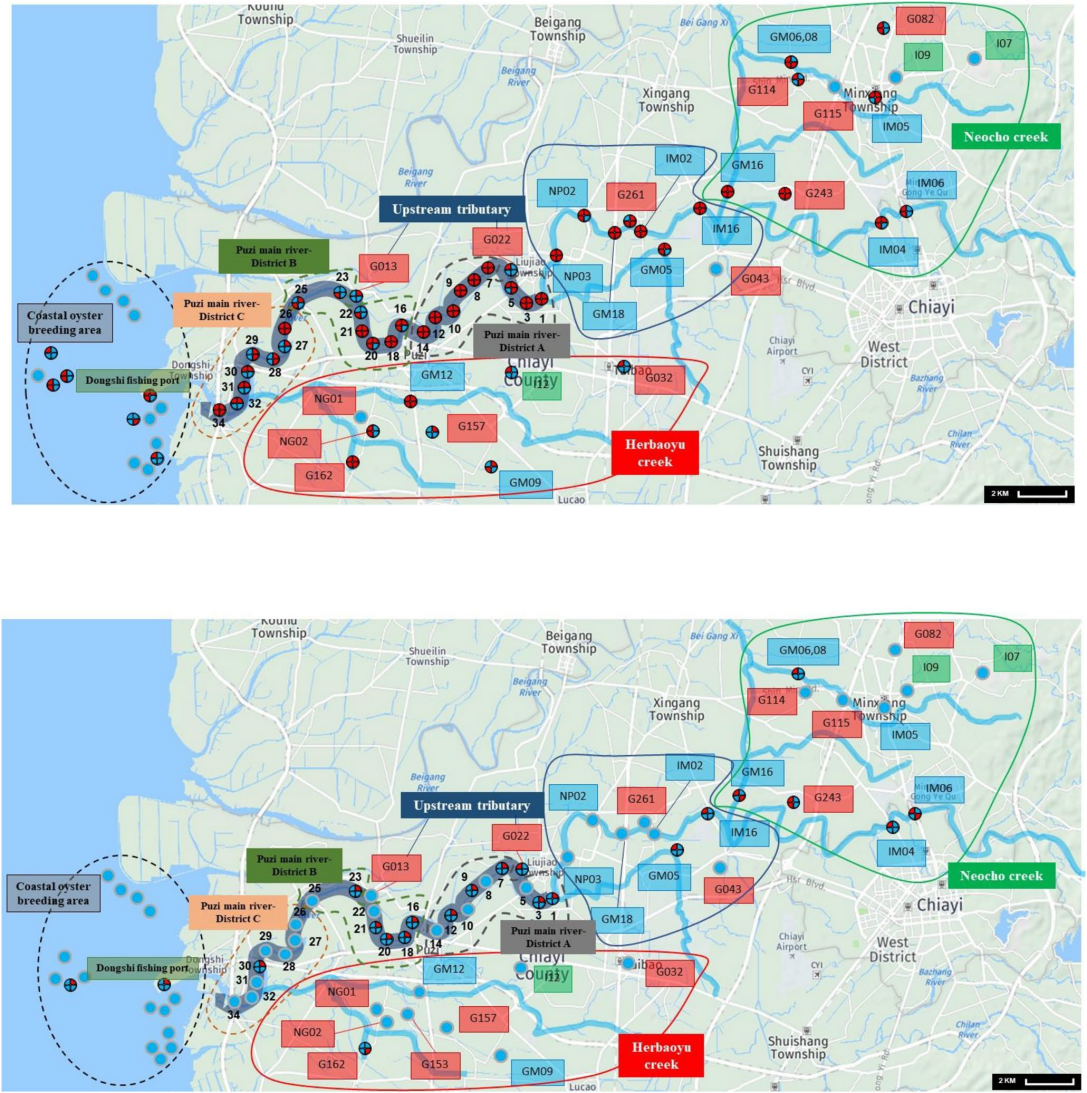
Distribution of (a) adenovirus (AdV) and (b) norovirus (NoV) hotspots in the Puzi River, its tributaries, Dongshi fishing port and Coastal oyster breeding area. Note: 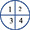 Pie chart denotes the seasonal occurrence of virus; 1‐ Autumn; 2‐ Winter; 3‐ Summer; 4‐ Spring; 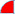 AdV/NoV positive; 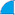 AdV/NoV negative.

The distribution of AdV hotspots in the Puzi River and surrounding sampling areas is summarized in Figure 1a. Area A of the Puzi River exhibited the highest prevalence of AdVs (97.2%), which may be related to local water pollution from various sources. The second highest prevalence of AdVs was in upstream tributaries (UT‐67.5%), followed by area B of the Puzi River (64.3%). The detection frequency of AdVs in area C of the Puzi River was 59.4%. Meanwhile, the AdV detection rates in the Neocho creek and Herbaoyu creek of the Puzi River were 52.3% and 46.9%, respectively. The increased rate of detection in these creeks may be due in part to the outflow of LFWC and MWTPC. The lowest AdV detection rate (16.7%) was recorded in the coastal oyster breeding area of the estuary and offshore of the Puzi River.

AdVs were detected in all water samples of areas A, B, and C of the Puzi River. The water samples collected from five sites in the tributary did not contain AdVs across four seasons. The hotspot distribution of AdVs was dense at the downstream outlet of the river, which can be attributed to the discharge points of LFWC and MWTPC near the Puzi River and tributaries. Moreover, the human settlement was dense at the upstream outlet. Thus, human and animal fecal contamination may lead to an increased prevalence of AdVs in these areas. However, the hotspot distribution of AdVs was sparse in UT and coastal oyster breeding areas because these areas are sparsely inhabited by humans.

The geographical distribution of NoV hotspots in the Puzi River and surrounding sampling areas is shown in Figure 1b. The prevalence of NoVs was the highest in the Neocho creek (18.2%), followed by area B (17.9%) and area A of the Puzi River (16.7%). The NoV detection rate was low in UT (7.5%) and Herbaoyu creek (3.1%). Similarly, the prevalence of NoVs was low in the coastal oyster breeding area (3.3%) and area C of the Puzi River (3.1%). In contrast, the distribution of NoV hotspots was dense in areas A and B of the Puzi River. However, the prevalence of NoVs markedly decreased near the downstream outlets (area C of the Puzi River and coastal oyster breeding area).

### 3.3 Genotyping and Phylogenetic Analysis of Adenovirus and Norovirus

In this study, 140 AdV‐positive samples were subjected to nucleic acid sequencing and species identification. Various genotypes of AdVs may be present in aquatic environments. HAdVs and PAdVs were predominant in the Puzi River and its tributaries, Dongshi fishing port, and oyster breeding area. The following five serotypes were the most prevalent: A species HAdV serotype 12 and serotype 31, F species HAdV serotype 41, A species PAdV serotype 3, and C species PAdV serotype 5 (Figure S3 in Supporting Information S1). The frequency of various HAdV and PAdV serotypes were examined (Table 2).

**Table 2:** Distribution of Various Adenovirus (AdVs) Genotypes in the Puzi River and Its Tributaries, the Dongshi Fishing Port, and the Coastal Oyster Breeding Area

AdVs genotypes	Puzi River water *n* = 96	Tributaries			Dongshi fishing port water *n* = 12	Oyster breeding area water *n* = 60
		UT *n* = 52	LFWC *n* = 52	MWTPC *n* = 12		
HAdVs 41	39	25	7	0	3	3
HAdVs 31	2	0	0	0	0	0
HAdVs 12	0	1	0	0	0	0
PAdVs 5	13	7	5	0	1	0
PAdVs 3	0	0	1	0	0	0

*Note*. The absence of all measured HAdVs and PAdVs in the MWTPCs samples. LFWC, Livestock farm wastewater channel; MWTPC, Municipal wastewater treatment plant channel; UT, Upstream tributaries.

The prevalence of HAdV genotypes was high in all sampling sites except the MWTPC sites. In particular, HAdV serotype 41 was the most prevalent in the Puzi River sampling sites, followed by PAdV serotype 5. HAdV serotype 31 was detected only in the Puzi River. Additionally, HAdV serotype 12 and PAdV serotype 3 were detected only in UT and LFWC, respectively. Moreover, both PAdV serotypes 3 and 5 were detected only in LFWC. But none of the serotypes was detected in MWTPC, which may be due to the higher efficiency of the water disinfection system installed in MWTPC. Additionally, the Dongshi fishing port was detected only HAdV serotype 41 and PAdV serotype 5.

The GI and GII genogroups of NoVs were prevalent in 33 positive samples from the Puzi River and its tributaries, Dongshi fishing port, and oyster breeding area. Nine genotypes (NoVs GI 2, 4, 5, 9; GII 2, 4, 13, 17, and Oyster/GII) of NoVs were prevalent in the samples, except MWTPC (Figure S4 in Supporting Information S1). The frequency of various NoV genotypes was determined (Table 3). GII.4 exhibited the highest detection frequency. However, GII.4 was not detected in the Dongshi fishing port and oyster breeding area. GII.17 was detected only in the Puzi River, while Oyster/GII was detected in all the sampling sites, except MWTPC. Additionally, GII.2 was detected only in the UT and LFWC. The lowest prevalence was exhibited by the genotypes GI.4, 5, and 9, which exhibited sporadic prevalence. GI.4 was detected only in the Dongshi fishing port and oyster breeding area. But none of the NoV genotypes were detected in MWTPC, which may be due to the higher efficiency of the water disinfection system installed in MWTPC.

**Table 3:** Distribution of Norovirus (NoV) Genotypes in the Puzi River and Its Tributaries, the Dongshi Fishing Port, and the Coastal Oyster Breeding Area

NoVs genotypes	Puzi river *n* = 96	Tributaries			Dongshi fishing port *n* = 12	Oyster breeding area *n* = 60
		UT *n* = 52	LFWC *n* = 52	MWTPC *n* = 12		
NoVs GI.2	0	3	0	0	0	0
NoVs GI.4	0	0	0	0	1	1
NoVs GI.5	1	0	0	0	0	0
NoVs GI.9	1	0	0	0	0	0
NoVs GII.2	0	3	2	0	0	0
NoVs GII.4	4	3	0	0	0	0
NoVs GII.13	3	0	0	0	0	0
NoVs GII.17	6	0	0	0	0	0
NoVs oyster/GII	1	2	2	0	1	1

*Note*. LFWC, Livestock farm wastewater channel; MWTPC, Municipal wastewater treatment plant channel; UT, Upstream tributaries.

### 3.4 Water Quality Analysis

Statistical analysis was performed to determine the correlation between the presence of AdVs and NoVs and the water quality indicators. The results of nonparametric tests for AdVs (Mann‐Whitney test) are shown in Table 4. The presence of AdVs was significantly correlated with heterotrophic bacterial count, total coliforms, *E. coli*, turbidity, salinity, and dissolved oxygen. In particular, AdVs were positively correlated with increased heterotrophic bacterial count, total coliforms, turbidity, and dissolved oxygen. In contrast, AdVs were negatively correlated with *E. coli* counts and salinity. The prevalence of AdVs was not correlated with the temperature and pH of water.

**Table 4:** Nonparametric Statistical Analysis of the Presence and Absence of AdVs in Relation to Water Quality Parameters From December 2015 and November 2016

Water quality indicators	Mann‐Whitney U test	AdVs—Positive samples (*n* = 85)			AdVs— Negative samples (*n* = 84)		
		Median	Q1	Q3	Median	Q1	Q3
Heterotropic plate count (CFU/ml)^a^	*P* < 0.01	92,893.7	7,383.33	119,385	43,284.58	33.33	19,100
Total coliform (CFU/100 ml)^a^	*P* < 0.01	1,824.54	221.42	1,992.5	750.38	0	172.33
*Escherichia coli* (CFU/100 ml)^a^	*P* < 0.01	2,034.22	4	98.5	47,135.1	0	3.33
Water temperature (°C)	*P* = 0.99	27.65	19.93	31.6	27.13	25.65	31.08
pH	*P* = 0.23	8.22	7.88	8.66	8.39	7.93	8.91
Turbidity (NTU)^a^	*P* < 0.01	50.09	23.4	67.7	15.85	4.28	21.60
Salinity (%)^a^	*P* < 0.01	7.68	0.44	13.67	21.75	14.94	31.74
Dissolved oxygen (mg/ml)^a^	*P* < 0.01	7.28	1.02	2.79	4.69	2.53	6.69

*Note*. Q1 for first quartile; Q3 for third quartile.

^a^ Water quality parameters that were significantly (*p* < 0.01) correlated with AdVs.

The results of nonparametric tests for NoVs (Mann‐Whitney test) are shown in Table 5. The prevalence of NoVs was correlated with the temperature, pH, and dissolved oxygen levels of water. In particular, the prevalence of NoVs was positively correlated with increased pH and decreased dissolved oxygen and temperature of the water. The prevalence of NoVs was not correlated with heterotrophic bacterial count, total coliforms, *E. coli*, turbidity, and salinity of water.

**Table 5:** Nonparametric Statistical Analysis of the Presence and Absence of NoVs in Relation to Water Quality Parameters From December 2015 and November 2016

Water quality indicators	Mann‐Whitney U test	NoVs—Positive samples (*n* = 156)			NoVs—Negative samples (*n* = 13)		
		Median	Q1	Q3	Median	Q1	Q3
Heterotropic plate count (CFU/ml)	*P* = 0.02	105,281	21,750	131,416.5	66,978.34	270	49,610
Total coliform (CFU/100 ml)	*P* = 0.07	1,214.11	671	1,131	1,322.54	3.5	1,327.75
*Escherichia coli* (CFU/100 ml)	*P* = 0.56	10	4	12	25,503.48	0	90.83
Water temperature (°C)^a^	*P* < 0.01	19.14	18.94	19.53	28.08	26.77	31.57
pH^a^	*P* < 0.01	9.05	8.48	9.39	8.25	7.9	8.7
Turbidity (NTU)	*P* = 0.26	2.14	1.07	3.21	31.18	6.67	43
Salinity (%)	*P* = 0.05	6.84	0.47	1.47	15.38	0.59	29.3
Dissolved oxygen (mg/ml)^a^	*P* < 0.01	1.93	0.86	1.58	6.32	1.58	5.69

*Note*. Q1 for first quartile; Q3 for third quartile.

^a^ Water quality parameters that were significantly (*p* < 0.01) correlated with NoVs.

## 4 Discussion

Taiwan, which is frequently affected by typhoons, has frequent thunderstorms in spring and summer (Fakour et al., 2016). These extreme weather events may be associated with the spread of various human pathogens (Webster et al., 2005). Thus, there is a need to examine the prevalence and seasonal distribution of pathogens, especially viruses, in aquatic ecosystems. Additionally, the discharges from livestock farms and municipal wastewater treatment plants may be a major source of contamination in the Puzi River and its tributaries. The viruses may be discharged from the animal/municipal wastewater treatment plants to natural water resources even after processing the wastewater, which may increase the risk of infection through contact with the recreational water. This study demonstrated that AdVs were the predominant viruses in the water samples. AdVs, which are non‐enveloped viruses, are frequently associated with upper respiratory tract syndromes, as well as with gastrointestinal, ophthalmologic, genitourinary, and neurological diseases (Flomenberg et al., 2020). The prevalence of AdVs was the highest in the Puzi River, followed by its tributaries, the Dongshi fishing port, and the coastal oyster breeding area. Previous studies have reported that HAdVs were prevalent in the Puzi River with the highest recorded detection rate of 87.5% (Tao et al., 2016). The AdV detection rate in water bodies may be influenced by geographical area, climatic conditions, and the type of detection method used (Huang et al., 2015; Rigotto et al., 2010). Natural disasters, such as heavy rainfall and runoff can be a source of AdV contamination in rivers. Additionally, river water may be contaminated with AdVs through wastewater discharge and urban runoff (Williamson et al., 2011). Furthermore, human and animal fecal contamination and anthropogenic activities may contribute to the AdV contamination of river water (Fong & Lipp, 2005; Hundesa et al., 2009). The prevalence of AdV was the highest in winter, followed by autumn. The studies conducted in Greece (Glafkos River) and Sweden (Umealven River) reported an increased prevalence of AdVs in the winter season (73% and 60%, respectively) (Rusinol et al., 2014). The prevalence of AdVs in winter is higher than that in summer as the spread of AdVs is inversely correlated with temperature (du Prel et al., 2009). Moreover, AdVs are reported to be stable at high humidity levels and exhibit an improved survival rate (80%). In contrast, the survival rate of AdVs is only 50% at low humidity levels (Davis et al., 1971). These findings explain the increased prevalence of AdVs in winter. In this study, AdVs were detected throughout the study period because the non‐enveloped viruses can survive throughout the year (Price et al., 2019). The prevalence of AdVs in the Puzi River sample was higher than that in the tributary and seawater samples. This was consistent with the results of a previous study, which reported that the prevalence of AdVs in the freshwater (41.1%) was higher than that in the seawater (27.4%) (Wyn‐Jones et al., 2011).

In this study, the prevalence of NoVs was the highest in the Puzi River, followed by its tributaries, the Dongshi fishing port, and the coastal oyster breeding area. NoVs are often detected in water bodies, including marine environments, rivers, lakes, water parks, and swimming pools (Félix et al., 2010; Koh et al., 2011; Podewils et al., 2007; Sartorius et al., 2007; Wyn‐Jones et al., 2011). Additionally, NoVs are one of the most common viral agents associated with waterborne diseases worldwide and can potentially cause acute viral gastroenteritis in people of all age groups (Wyn‐Jones et al., 2011). A previous study reported that the detection rate of NoVs ranged from 53%–81% and that the prevalence of NoVs was dependent on the level of water contamination (Mans et al., 2013). Genetic diversity and high mutation rate may contribute to the prolonged survival of NoVs in water (Hansman et al., 2006). The prevalence of NoVs significantly differs between seasons. The changes in natural events and environmental conditions are associated with the seasonality of viral spread. The prevalence of NoVs was the highest in the winter season, followed by autumn. The epidemic characteristics of NoVs are consistent every year although the peak incidence is reported during winter (Verhoef et al., 2008). Previous studies in South Korea, Spain, and Japan have reported that the prevalence of NoVs was the highest in the winter season, followed by autumn and spring (Kim et al., 2016; Kitajima et al., 2010; Pérez‐Sautu et al., 2012). NoV epidemics are high in the cold months in Northern Hemisphere countries (Marshall & Bruggink, 2011). The increased humidity and low water temperature in winter may facilitate an increased prevalence of virions. In this study, the prevalence of NoVs was low in the tributary in summer, which is rarely associated with off‐season spread (Rohayem, 2009).

Genotyping and phylogenetic analysis revealed that HAdV was the predominant genotype in the water samples, followed by PAdV. The origin of HAdV contamination is anthropogenic activities. Moreover, the spread of HAdV may be sporadic throughout the season, which is independent of rainfall (Choi & Jiang, 2005). Enhanced precipitation and increased runoff may be associated with a high load of HAdVs (Mackowiak et al., 2018). HAdV species F serotype 41 was the most frequently detected virus in the Puzi River. Compared with other serotypes, HAdV serotype 41 is highly persistent in the natural environment. Thus, individuals in contact with the river water may be at an increased risk of infection with HAdV serotype 41. Previous studies have demonstrated that HAdV serotypes 40 and 41 are the predominant serotypes in various water bodies, such as rivers, dam water, or drinking water (Chapron et al., 2000; Vergara et al., 2016). Additionally, HAdV serotype 41 is the most commonly detected HAdV in wastewater and surface water in the United States, Greece, Spain, Luxembourg, and Europe (Bofill‐Mas et al., 2006; Fong et al., 2010; Kokkinos et al., 2010; Ogorzaly et al., 2015; Wyn‐Jones et al., 2011). Compared with other AdV serotypes, HAdV serotype 41 may exhibit increased persistence in natural environments. HAdV serotype 41 is the etiological agent for most cases of gastroenteritis in children. The prevalence of HAdV serotypes 12 and 31 was low in the UT and Puzi River, respectively. The likely sources of these serotypes are the diluted raw sewage and primary effluent discharge from surrounding wastewater treatment plants. These serotypes cause diarrhea and acute hemorrhagic cystitis in humans, especially in children (Formiga‐Cruz et al., 2002; Rames et al., 2016). The present study analyzed the river, tributaries, fishing port and coastal oyster breeding area water for viral contamination. Future studies should be conducted to include more direct characterization of both human (sewage treatment plants effluent) and domestic animal (livestock farm water) sources of pollution within this region. That will provide a direct measure of the above‐said pollution sources. However, the introduction of viral sources from pets, animals and wildlife may occur.

In this study, PAdV serotypes 3 and 5 were detected only in the LFWC samples. Animal feces and urine disseminated from the LFWC may contaminate the tributary water via runoff or drainage. These serotypes are potential indicators of swine fecal contamination (Hundesa et al., 2006). Additionally, PAdVs are highly prevalent in slaughterhouse wastewater and are associated with subclinical infection (Hundesa et al., 2006). Moreover, PAdVs cause gastrointestinal disease, diarrhea, and multifactorial respiratory disease in swine (Fongaro et al., 2015). The prevalence of PAdVs was low in this study, which may be attributed to the increased dilution of outflow from LFWC. PAdV serotype 5 was also detected in the Puzi River and Dongshi fishing port, which may be likely attributed to the outflow from LFWC. Hence, this study demonstrated that the sample sites (Puzi River, tributaries, Dongshi fishing port, and oyster breeding area) were associated with both human and animal fecal contamination. PAdVs were not detected in the coastal oyster breeding area, which indicated that it was free from animal fecal contamination. This finding is important as molluscan shellfish are particle feeders and are known to concentrate viruses. HAdVs and PAdVs were not detected in the MWTPC samples, which may be due to the high efficiency and performance of the wastewater treatment plants. This finding is very important as this type of technology for waste disinfection can be successfully applied to other pollution sources such as swine waste.

Among NoVs, the prevalence of GII was higher than that of GI in this study. GII NoVs are responsible for most viral diseases in humans. The GI group cannot survive in water at temperatures above 4°C. This explains the decreased detection frequency of GI in the sampling sites (Skraber et al., 2009). GII.4, which was detected only in the Puzi River and UT, causes gastroenteritis in humans and is one of the genotypes frequently detected in human clinical samples (Skraber et al., 2009). Consistently, previous studies have reported that the genotypes GII.4 (79%) and GII.17 (64%) were predominant in Japan and Kenya, respectively (Iwai et al., 2009; Kiulia et al., 2014). GI.2, which is also associated with waterborne disease outbreaks, was detected only in UT. This genotype is frequently detected in sewage and surface water (Kazama et al., 2016). GI.4, which was detected only in the Dongshi fishing port and coastal oyster breeding area, is detected in sewage water and is associated with foodborne NoV outbreaks in many countries (Kazama et al., 2016). The presence of GI.4 in the Dongshi fishing port may be related to discharges from fishing vessels within this region.

In this study, GII.17, which was detected in the Puzi River, is a novel genotype that is reported to cause severe gastroenteritis outbreaks in countries, such as China, Taiwan, and Japan (Cheng et al., 2017; Lu et al., 2015; Matsushima et al., 2015). In early 2015, the outbreak of GII.17 led to more than 200 cases of gastroenteritis among students with a severe infection case (Cheng et al., 2017). GII.17 was detected in aquatic environments from late 2015 to early 2016, which indicated that the prevalence of this genotype increased and that there is a need for continuous surveillance. GI and GII were detected in the Puzi River, UT, and LFWC. The coexistence of both genotypes may facilitate the emergence of novel intergenogroup recombinants. NoVs may be transported from the contamination source to the river and its tributary. River water contaminated with NoVs may be a potential vector for viral transmission during recreational activities. This study demonstrated that the Puzi River, UT, and surrounding sites were contaminated with clinically relevant NoV genotypes (GI.2, GII.4, and GII.13), which may have been attributed in part to the presence of Puzi hospital. However, NoV genotypes were not detected in MWTPC, which may be attributed to the high performance of municipal wastewater treatment plants.

Correlation analysis revealed that the presence of AdVs is correlated with many water quality indicators. The presence of AdVs was associated with high heterotrophic bacterial counts, total coliforms, turbidity, and dissolved oxygen in the water. A previous study reported that the prevalence of HAdVs in water is significantly associated with various physicochemical parameters (Silva et al., 2011). AdVs have been proposed as water quality indicators for human or animal fecal pollution (Hundesa et al., 2006; Rames et al., 2016). Some of the water quality indicator bacteria and coliforms may be generated from many sources including humans, pets, domestic animals and wildlife sources which may concentrate in riverbed sediments. These bacteria are often flushed out by rainwater runoff and transported to rivers (Desmarais et al., 2002; Roll & Fujioka, 1997). The prevalence of NoVs was associated with increased pH and decreased dissolved oxygen and temperature. The survival rate of NoVs depends on the temperature of the water and it can survive for a prolonged duration in cool water (Allwood et al., 2003). The increasing global temperature associated with climate change may have an impact on NoVs levels in many aquatic and coastal ecosystems around the world.

## 5 Conclusions

This study investigated the prevalence, distribution, and genotypes of AdVs and NoVs in environmental water samples and their correlation with water quality. AdVs were more prevalent than NoVs in the Puzi River and its tributaries (UT, LFWC, and MWTPC). This suggested that AdVs are a potential water quality indicator. The predominance of HAdV species F serotype 41 and NoV GII.4 in the sampling sites indicate the need for viral quantification studies in the future. NoV GII.17, which was detected in the sampling site, can be a potential public health challenge. The source of AdVs and NoVs may include the outflow of LFWC and urban‐runoff into the river ecosystem. The identification of natural events and anthropogenic activities can aid in determining the source of contamination for remediation purposes. Future studies must explore the potential threats associated with AdVs and NoVs from river and coastal ecosystems.

## Conflict of Interest

The authors declare no conflicts of interest relevant to this study.

## Supporting information

Supporting Information S1

## Data Availability

The data presented in this study are available on request from the corresponding author and also are available on figshare website (https://doi.org/10.6084/m9.figshare.16382274.v1).
