# Supplementary material for: Prevalence, Distribution, and Genotypes of Adenovirus and Norovirus in the Puzi River and Its Tributaries and the Surrounding Areas in Taiwan
Source: Geohealth. 2021 Dec 1;5(12):e2021GH000465. doi: 10.1029/2021GH000465 (PMC8686652; doi:10.1029/2021GH000465)
Supplement: Supplementary file 1 — Supporting Information S1 [file GH2-5-e2021GH000465-s001.pdf]

Supplementary materials for

Prevalence, distribution, and genotypes of adenovirus and norovirus in the Puzi River and its tributaries and the surrounding areas in Taiwan

Viji Nagarajan[a1], Jung-Sheng Chen[b], Bing-Mu Hsu[a*], Gwo-Jong Hsu[c1], Jiun-Ling Wang [d1] and Bashir Hussain[a,e]

[a] Department of Earth and Environmental Sciences, National Chung Cheng University, Chiayi County, Taiwan

[b] Department of Medical Research, E-Da Hospital, Kaohsiung, Taiwan

[c] Division of Infectious Diseases, Ditmanson Medical Foundation, Chia-Yi Christian Hospital, Chiayi City, Taiwan

[d] Department of Internal Medicine, National Cheng Kung University Hospital, Tainan, Taiwan

[e] Department of Biomedical Sciences, National Chung Cheng University, Chiayi County, Taiwan

*Corresponding author: Bing-Mu Hsu (bmhsu@ccu.edu.tw)

[1] Contributed equally to this work

Contents of this file

This file contains figure S1 to S4

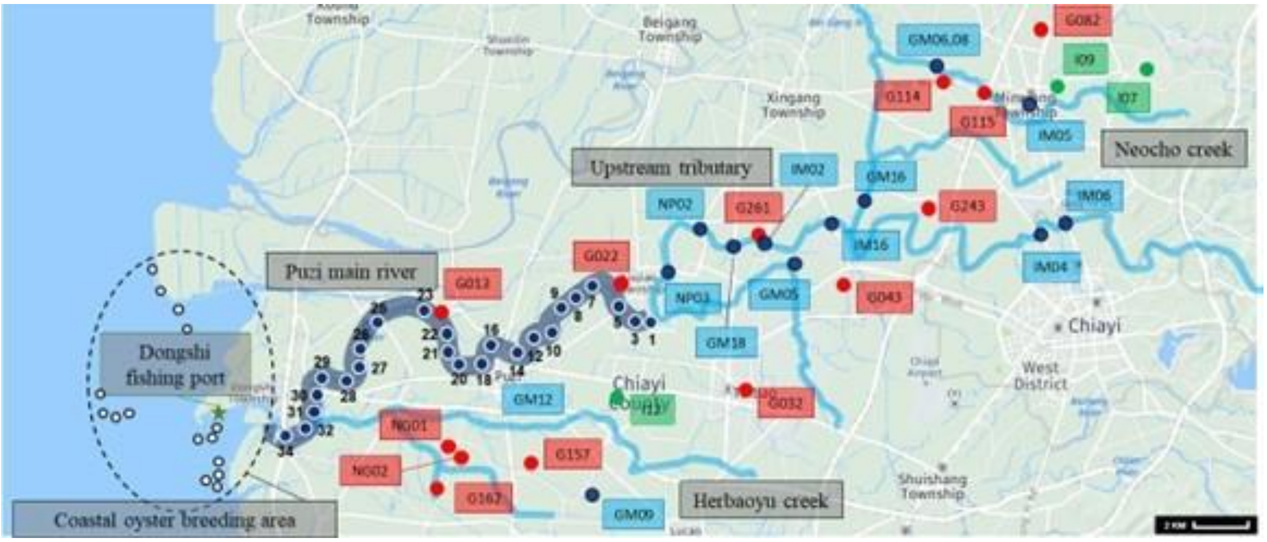

Figure S1. Location and distribution of sampling points in the study area

Note: ◉ Puzi River; ● Upstream tribuatary; ☆ Dongshi fishing port; ○ Coastal oyster breeding area; ● Livestock farm wastewater; ● Municipal wastewater treatment plant channel.

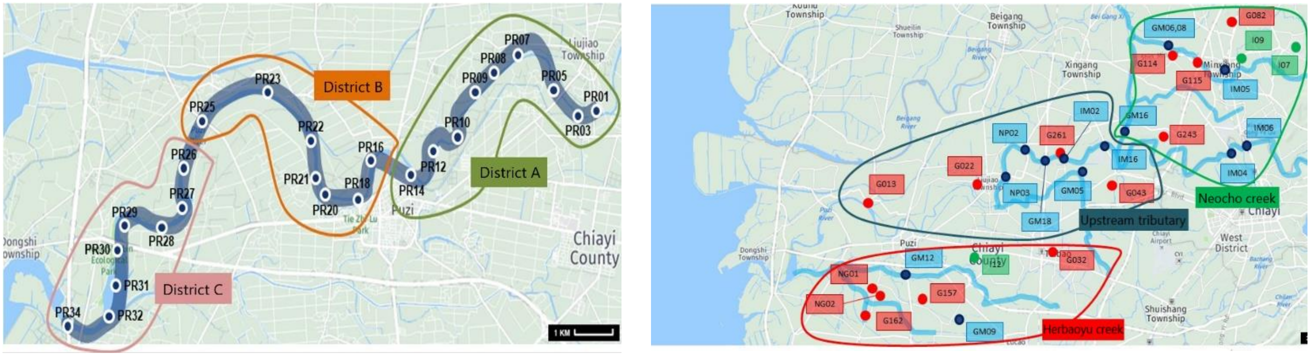

(a) **(b)**

Figure S2. Sampling locations in the (a) Puzi main river and (b) Upstream tributaries

Note: (a) ■ District A; ■ District B; ■ District C.

(b) ■ Herbaoyu creek; ■ Upstream tributary; ■ Neocho creek;

● Upstream tributaries; ● Livestock farm wastewater channel; ● Municipal wastewater treatment plant channel.

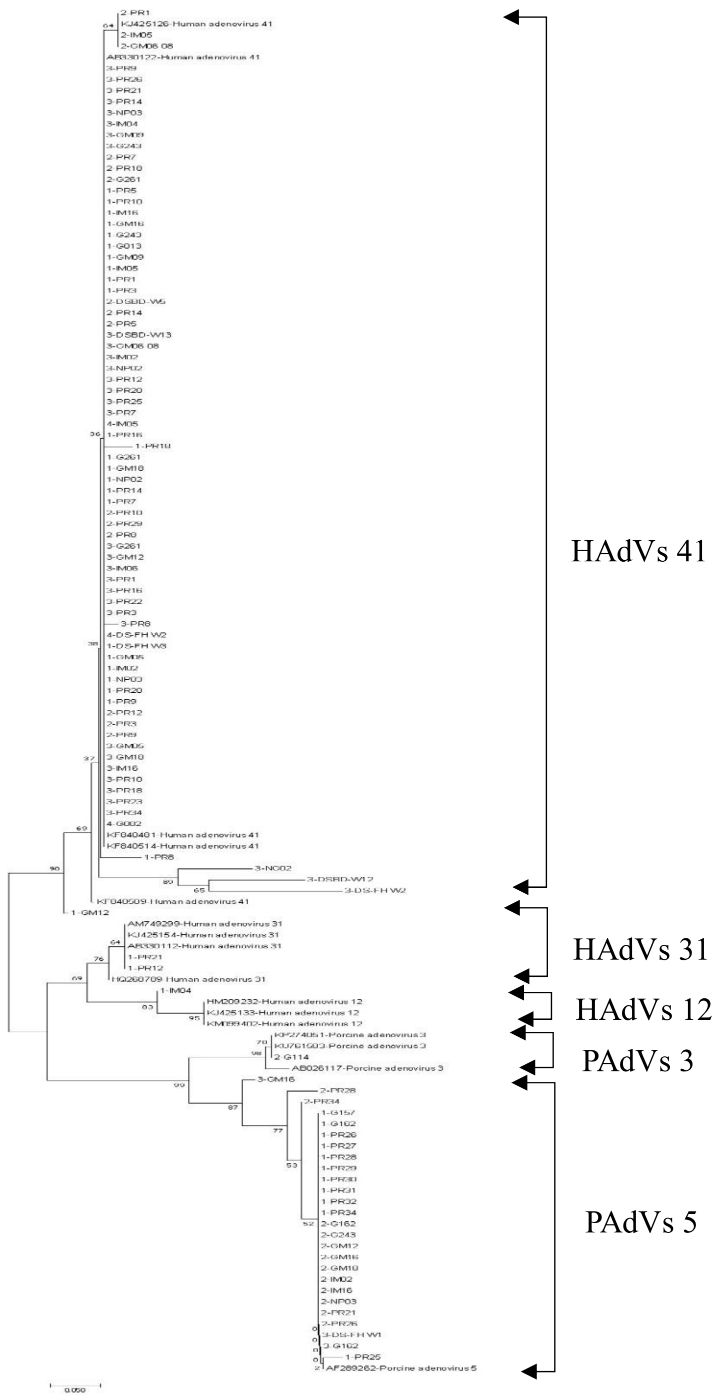

Figure S3. Genetic evolution analysis of AdVs in the Puzi river, its tributaries and near sampling sites

Note: The first number indicates the four seasons (1 winter; 2 spring; 3 summer; 4 autumn); the abbreviation of the station name- PR Puzi main river; IM, GM, G, I tributary water near the LFWC and MWTPC; FH the seawater sample of the Dongshi fishing port; DSBD Dongshi Budai coastal oyster breeding area; W water body sample plus number shows sampling point number.

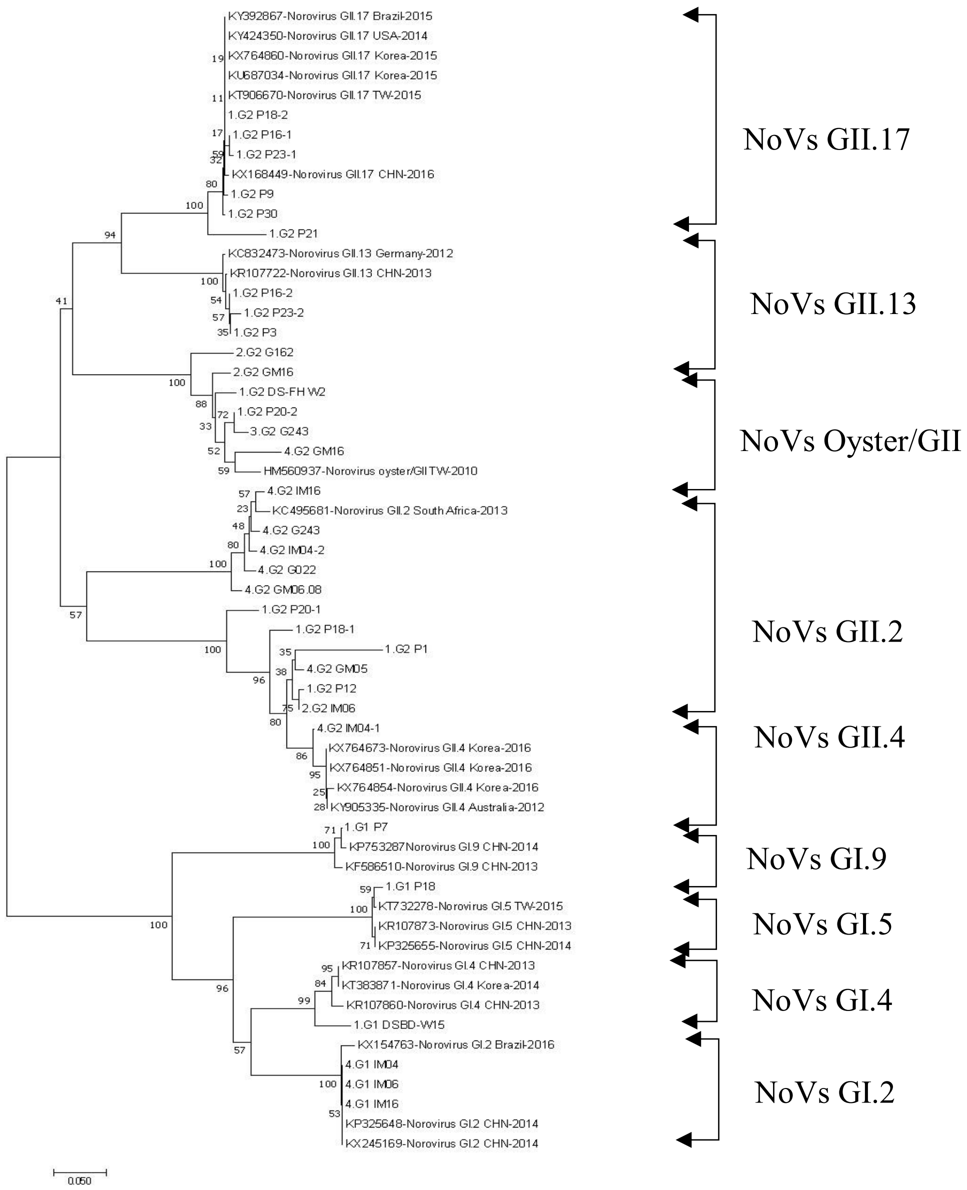

Figure S4. Genetic evolution analysis of NoVs in the Puzi river, its tributaries and near sampling sites

Note: The first number indicates the four seasons (1 winter; 2 spring; 3 summer; 4 autumn); the abbreviation of the station name- PR Puzi main river; IM, GM, G, I tributary water near the LFWC and MWTPC; FH the seawater sample of the Dongshi fishing port; DSBD Dongshi Budai coastal oyster breeding area; W water body sample plus number shows sampling point number.
